# The effect of age on full-field electroretinograms recorded with skin electrodes

**DOI:** 10.20407/fmj.2020-006

**Published:** 2020-12-16

**Authors:** Daisuke Samoto, Atsuhiro Tanikawa, Keita Suzuki, Hidenori Tanaka, Tadashi Mizuguchi, Yoshiaki Shimada, Masayuki Horiguchi

**Affiliations:** Department of Ophthalmology, Fujita Health University, School of Medicine, Toyoake, Aichi, Japan

**Keywords:** International Society for Clinical Electrophysiology of Vision, Electroretinography, Skin electrode, Aging, Photopic negative response

## Abstract

**Objectives:**

The aim of this study was to determine whether age correlates with amplitude and latency, when full-field electroretinography (ERG) is performed using skin electrodes. The ability of pulse reference power line noise reduction (PURE) to dampen the noise associated with the use of skin electrodes, was also investigated.

**Methods:**

ERG was performed on 77 eyes in 77 healthy subjects (mean age: 55.6±19.0 years; age range: 9 to 86 years). Subjects with –5D or higher myopia, Emery-Little grade III or higher cataracts, retinal disease, uveitis, glaucoma, ≤5 mm mydriasis, or a history of intraocular surgery other than cataract surgery, were excluded. The active, reference, and ground electrodes were placed on the lower eyelid, outer canthus, and earlobe, respectively. Responses were averaged 10 times for dark-adapted (DA) ERGs, and 32 to 64 times for light-adapted (LA) ERGs. Noise was removed using the PURE method.

**Results:**

The DA ERGs without PURE were so noisy that the amplitude or latency could not be determined, whereas those with PURE were comparatively quieter. ERG with PURE demonstrated a significant negative correlation between age and amplitude and a significant positive correlation between age and latency.

**Conclusions:**

We could record the measurable ERG waveforms with skin electrodes by using the PURE method, especially in fewer averaged conditions. It is suggested that skin electrode with PURE is suitable to examine the pathological ERGs, and other types of electrodes. It is recommended that the aging effect should be taken into consideration when pathological ERGs are evaluated.

## Introduction

The International Society for Clinical Electrophysiology of Vision (ISCEV) has established a standard protocol for electroretinography (ERG).^1^ It also describes stimulation methods and basic electrode technologies and recommends the use of facility-specific reference values for ERG. However, because some ERG parameters change with age, the reference values need to be age-adjusted.

Several studies of healthy subjects indicate that the amplitude determined via full-field ERG decreases with age, whereas the latency increases.^2–7^ In these studies, the ERG data were recorded using contact lens electrodes,^2,4,5^ Dawson-Trick-Litzkow (DTL) electrodes,^6–8^ or gold foil electrodes.^3^ However, no similar studies using skin electrodes have been reported.

According to the 2015 ISCEV protocol for full-field clinical ERG, ERG signal amplitude is lower with non-contact electrodes such as skin electrodes, than with contact electrodes such as contact lens, DTL, and gold foil electrodes, and thus may not be suitable to evaluate attenuated pathological electroretinograms.^1^ This is presumably because lower amplitudes are more susceptible to noise. Because noise can render data unusable, skin electrodes are considered unsuitable for acquiring ERG data at most facilities.

Pulse reference power line noise reduction (PURE) is a noise reduction method that synthesizes hum noise using the Fourier transform and removes it from the original waveform while referencing an AC power source.^9^ An ERG recorder equipped with PURE minimizes noise, even when using skin electrodes. In this study, we used a PURE-equipped ERG system to elicit ERGs from skin electrodes in healthy subjects. The correlation between these factors and age was determined. ERG was performed in accordance with the ISCEV guidelines and using the ERG recording setup of our hospital.

## Methods

ERG was performed on 77 eyes in 77 healthy subjects (mean age, 55.6±19.0 years; range: 9 to 86 years), of whom 40 were women and 37 were men. The corrected visual acuity was 1.0 or higher in all cases. We did not include subjects with –5D or higher myopia, Emery-Little grade III or higher cataracts,^10^ retinal and/or macular disease, chorioretinal atrophy, uveitis, glaucoma, mydriasis ≤5 mm, or a history of intraocular surgery other than cataract surgery. Twenty-one of the 77 eyes had an intraocular lens.

This study was conducted in accordance with the tenets of the Declaration of Helsinki and was approved by the Ethics Committee of Fujita Health University. All subjects were informed of the purpose of the study and the methods used and that they would not be disadvantaged if they decided not to participate in the study, after which we obtained consent for their participation in the study.

The 2015 ISCEV guidelines for full-field clinical ERG include six ERG protocols, each named according to the state of adaption (dark or light) followed by the stimulus intensity (cd-s/m^2^).^1^ In this study, four out of the six ERG protocols, namely dark-adapted (DA) 0.01, DA 3, light-adapted (LA) 3, and LA flicker ERG, were investigated. After 20 minutes of dark adaptation following mydriasis with 0.5% phenylephrine and 0.5% tropicamide eye drops, DA ERGs were elicited. After 10 minutes of light adaptation, LA ERGs were measured. All ERG responses were recorded using an ERG recorder (PuREC; Mayo Corp., Inazawa, Japan) with full-field stimulus. White light-emitting diodes were used as the light source for the light stimulus and the background light.

The skin electrodes were silver plate electrodes. The active, reference, and ground electrodes were placed on the lower eyelid, lateral canthus, and earlobe, respectively. The upper cut-off value for the band pass-filter was set at 500 Hz and the lower cut-off value at 0.3 Hz. Responses were amplified 10,000-fold and averaged 10 times for the DA ERGs and 32 to 64 times for the LA ERGs. Noise was removed using the PURE method.

**Figure 1:**
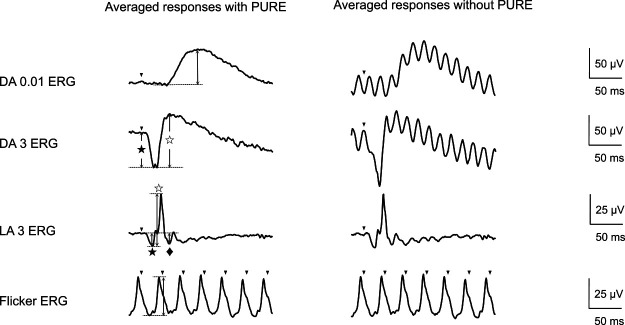
Representative electroretinography (ERG) waveforms recorded in a healthy subject using skin electrodes with or without pulse reference power line noise reduction (PURE) The waveforms shown are from a 22-year-old subject with no ophthalmological disease. Dark-adapted ERGs without PURE were so noisy that the amplitude or latency could not be determined, whereas those with PURE were much less noisy. DA: Dark-adapted, LA: Light-adapted, PURE: pulse reference power line noise reduction Arrowheads indicate the stimulus flash; ★: a-wave; ☆: b-wave; ◆: photopic negative response

The amplitude and latency of each ERG component were measured from the elicited waveforms. The amplitude of a- and b-wave was measured from the baseline to the a-wave trough, and from the a-wave trough to the b-wave peak, respectively. The DA 0.01 amplitude was measured from the baseline to the peak. The LA flicker ERG amplitude was measured from the trough to the peak. The ISCEV extended protocol for the photopic negative response (PhNR)^11^ recommends eliciting the PhNR by a red stimulus on a blue background; however, there are several reports in which PhNR was determined in the LA 3 ERG waveform.^12–18^ Ortiz et al.^18^ investigated two PhNR troughs in the LA 3 ERG: one with PhNR1 as a trough before the i-wave and the other with PhNR2 as a trough after the i-wave. Thus, the amplitude of PhNR1 as PhNR was determined from the baseline to the trough in the LA 3 ERG waveform. The latency of each component was measured from the flash to the peak of the wave (Figure 1, left). Median values were calculated in accordance with ISCEV recommendations, as were the 5th and 95th percentiles of the amplitude and latency.^1^ Correlations with age were determined via linear regression analysis. A p value <0.05 indicated significance of the standardized coefficient.

## Results

Figure 1 shows representative waveforms recorded using skin electrodes, with or without PURE, for comparison. The best corrected visual acuity was 1.0×S-3.5 D in the right eye and 1.0×S-3.25 D in the left eye, and the intraocular pressure was 12 mmHg in both eyes. No abnormalities were observed in the anterior segment, ocular media, or fundus. The DA ERGs were fewer averaged than the LA ERGs to prevent from the light adaptation. The DA ERGs without PURE were so noisy that the amplitude or latency could not be determined, whereas those with PURE were lesser noisy.

**Figure 2:**
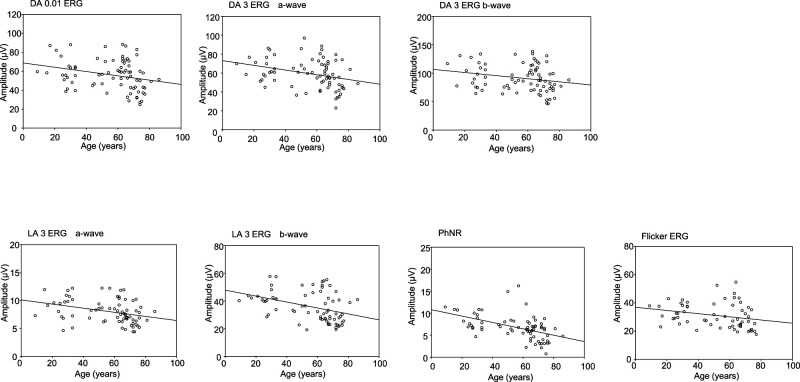
Scatter plots charting amplitude and age for each electroretinography component in the standard International Society for Clinical Electrophysiology of Vision protocol DA: Dark-adapted, LA: Light-adapted, PhNR: photopic negative response

Table 1 shows the results of a linear regression analysis, assessing the relationship between median amplitude in the 5th and 95th percentiles and age. All components (DA 0.01; DA 3 a-wave and b-wave; LA 3 a-wave, b-wave, and PhNR; and LA 30 Hz flicker) were significantly and negatively correlated with age. In scatter plots, the amplitude of the PhNR was moderately correlated with age, whereas that of the other components was weakly correlated (Figure 2 and Table 1).

**Figure 3:**
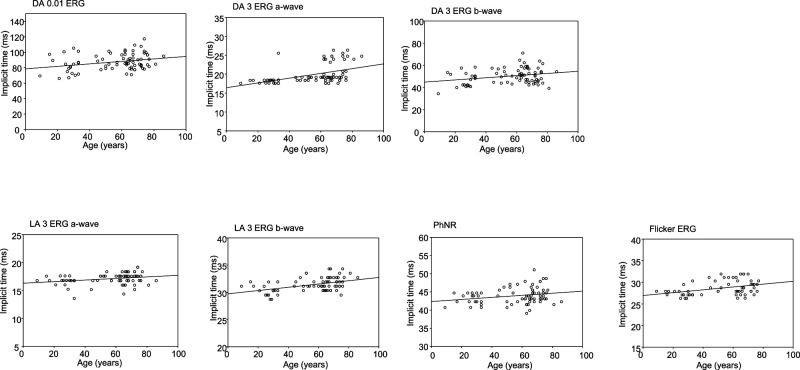
Scatter plots charting latency and age for each standard electroretinography component DA: Dark-adapted, LA: Light-adapted, PhNR: photopic negative response

Table 2 shows the results of a linear regression analysis assessing the relationship between median latency in the 5th and 95th percentile and age. All seven components were significantly and positively correlated with age. In scatter plots, DA 0.01, DA 3 b-wave, LA 3 a-wave, PhNR, and flicker were weakly correlated with age, whereas DA 3 a-wave and LA-3 b-wave was moderately correlated with age (Figure 3 and Table 2).

## Discussion

In this study, we used PURE to successfully reduce noise when performing ERG using skin electrodes. Consequently, we could establish the reference values such as the median value along with the 5th and 95th percentiles of the amplitude and latency of each ERG component in our setup, as per ISCEV recommendations.^1^ The PURE method synthesizes hum noise using the Fourier transform and removes it from the original waveform while referencing an AC power source. However, even when using PURE, mixing of responses with the electromyogram from the eyelids is unavoidable. Therefore, to reduce mixing, we increased the number of times the responses were averaged, so that it was approximately twice of that used when recording with corneal electrodes.

In our study, ERG was performed using skin electrodes. For all ERG components, amplitude was significantly and negatively correlated with age, whereas latency was significantly and positively correlated with age. These results are almost the same as those reported in previous studies using different types of electrodes.^2–7^ Skin electrodes with PURE may be suitable to evaluate pathological ERGs as well as other types of electrodes. It is also recommended to consider the aging effect on pathological ERG responses when they are compared with the reference values. The amplitude measured via ERG is a summation of the extracellular potentials of rod, cone, bipolar, ganglion, amacrine, horizontal, and Müller cells throughout the entire retina.^19,20^ Age-related decreases in the number of rod, bipolar, ganglion, and Müller cells^21–23^ and associated changes in signal transduction are thought to reduce the amplitude and delay the latency. However, it is difficult to capture changes in the extracellular potential in each cell type via ERG. This will require more detailed research.

The PhNR reflects the activity of retinal ganglion cells and their axons.^24^ The extended ISCEV protocol states that a red stimulus on a blue background is suitable for recording the PhNR because this stimulus combination yields a larger amplitude than the others.^11^ However, in this study, we used a white stimulus on a white background, which is the stimulus condition for LA 3 ERG. Nevertheless, a previous study using a red stimulus on a blue background reported results similar to ours (i.e., negative correlation of age with PhNR amplitude and positive correlation of age with PhNR latency).^25^

Miura et al. recorded flicker responses in patients with grade II and III cataracts using skin electrodes and a mydriasis-free RETeval device. In these patients, amplitude was significantly lower and latency was significantly longer than in patients with pseudophakia.^26^ It is possible that the inclusion of subjects with mild cataracts (Emery-Little grade II or lower) in our study may have affected our results.^27^ However, a strong stimulus (as used in ISCEV-standardized ERG) better reduces the effects of cataracts on latency than does a flicker stimulus (which is used in RETeval ERG).^28^ Therefore, we believe that mild cataracts did not significantly affect our results. Suzuki et al. performed S-cone ERG and LM-cone ERG on 31 patients (ages 25 to 91 years) with intraocular lenses and no retinal diseases and reported a significant age-related decrease in amplitude and the duration of latency.^5^ Therefore, even when the effects of cataracts are removed, correlations between aging and ERG amplitude and latency similar to ours are demonstrable.

Our study has the following limitations. First, because our sample size was relatively small, the values reported here (median amplitude and latency and 5th and 95th percentiles of each ERG components) might not apply to larger cohorts. Second, because some subjects in our study had mild cataracts, an effect of cataracts on our results cannot be completely eliminated. Ideally, only subjects with intraocular lenses should be studied in this context, as in the Suzuki et al. study.^5^ However, few young people have intraocular lenses, which complicates subject recruitment.

In this study, we successfully performed ISCEV-standardized ERG on healthy patients using skin electrodes. The waveforms were good, and noise was minimal presumably owing to use of PURE. For each ERG component, age was negatively associated with amplitude and positively with latency. In the future, it will be necessary to investigate these relationships in a larger cohort without cataracts or with a greater awareness of the effects of cataracts.

## Supplementary Material

PDF-Japanese

## Figures and Tables

**Table1:** Correlation between median amplitude in the 5th and 95th percentiles and age

		Median (μV)	5th percentile (μV)	95th percentile (μV)	Standardized coefficient	P value
DA 0.01		56.1	29.8	86.4	–0.269	0.018
DA 3	a-wave	58.7	35.4	85.4	–0.313	0.006
	b-wave	87.6	54.5	132.5	–0.224	0.049
LA 3	a-wave	7.9	4.8	11.9	–0.330	0.005
	b-wave	33.5	21.8	54.5	–0.386	<0.001
	PhNR	6.7	2.9	11.4	–0.499	<0.001
LA 30 Hz flicker		29.5	19.8	45.3	–0.256	0.049

DA: Dark-adapted, LA: Light adapted, PhNR: photopic negative response

**Table2:** Correlation between median latency in the 5th and 95th percentiles and age

		Median (ms)	5th percentile (ms)	95th percentile (ms)	Standardized coefficient	P value
DA 0.01		85.4	70.5	104.5	0.287	0.001
DA 3	a-wave	19.2	17.6	25.3	0.465	<0.001
	b-wave	50.3	40.7	62.0	0.269	0.018
LA 3	a-wave	17.2	15.2	18.4	0.270	0.022
	b-wave	31.1	29.5	33.5	0.449	<0.001
	PhNR	43.9	40.7	47.9	0.242	0.040
LA 30 Hz flicker		27.9	26.3	31.9	0.383	0.003

DA: Dark adapted, LA: Light-adapted, PhNR: photopic negative response
