# Supplementary material for: The effect of age on full-field electroretinograms recorded with skin electrodes
Source: Fujita Med J. 2020 Dec 16;7(4):117–21. doi: 10.20407/fmj.2020-006 (PMC8761819; doi:10.20407/fmj.2020-006)
Supplement: Supplementary file 1 — PDF-Japanese [file fmj-7-117-s001.pdf]

**抄録**

目的: 皮膚電極を使って正常被験者から記録した全視野網膜電図(electroretinogram: ERG)の振幅や潜時と年齢との相関、そして皮膚電極におけるpulse reference power line noise reduction（PURE法）によるノイズ軽減効果も検討する。

方法: 対象は正常者 77 名 77 眼（9〜86 歳、平均 55.6 ± 19.0 歳）。-5D 以上の近視、Emery-Little 分類 3 度以上の白内障、網膜疾患、ぶどう膜炎、緑内障、白内障を除く内眼手術の既往、5mm 以下の散瞳不良は除外した。関電極は下眼瞼、不関電極は外眼角、接地電極は耳朶に設置した。反応の加算回数は暗順応下の ERG では 10 回、明順応下での ERG では 32 回から 64 回行った。

結果: PURE 法なしの暗順応下の ERG ではノイズが大きいため振幅や潜時が計測できなかったが、PURE 法を用いるとノイズは比較的小さかった。PURE 法を用いた ERG では年齢と振幅は有意な負の相関を示し、年齢と潜時は有意な正の相関を示した。

結論: PURE 法により皮膚電極からノイズの少ない波形を記録できた。特に少ない加算回数の場合に PURE 法は有効であった。PURE 法を用いることで皮膚電極でも、他の電極と同様に疾患の ERG を評価できることが示唆された。またその場合には年齢による振幅や潜時の変化も考慮すべきである。



Keywords

International Society for Clinical Electrophysiology of Vision, electroretinography, Skin electrode, Aging, photopic negative response

## 本文

序論

International society for clinical electrophysiology of vision: ISCEV では electroretinogram: ERG の標準化したプロトコルを提唱している[1]。そこでは刺激方法や電極等についても述べられおり、各施設での正常データの取得が勧められている。

正常者の full-field ERG と年齢との相関についてはいくつか報告[2-7]があり、振幅は加齢に伴い減少し、潜時は延長する。これらの報告はコンタクトレンズ電極[2, 4, 5]や Dawson-Trick-Litzkow (DTL)電極[6-8]、金箔電極[3]などから記録した ERG のデータで、皮膚電極から記録した ERG の報告はない。

2015 年の ISCEV standard ERG update では皮膚電極について "The ERG signal amplitude is lower with non-contact electrodes"、"may not be suitable to evaluate attenuated pathological ERGs"と言及されている[1]。この理由として、皮膚電極による ERG は他の電極に比べて振幅が小さい上に、大きなノイズが混入しやいため、施設での正常データを取得するのには適さなかったからと考えられる。

Pulse reference power line noise reduction system: PURE 法は戸田が開発したノイズ除去方法で、交流電源を参照しながら、フーリエ変換を用いてハムノイズを合成し、元波形から除去する[9]。このノイズ除去方法を搭載した ERG 記録装置を用いることで皮膚電極からもノイズの少ない良好な反応を記録することが可能となった。今回我々はこの ERG 記録装置を用いて正

常者から皮膚電極でISCEVの標準化されたERGを記録し、当院のERG記録条件における振幅、潜時の正常範囲を求め、年齢との相関を検討した。

方法

対象は正常者77名77眼、男性37眼、女性40眼（9～86歳、平均55.6 ± 19.0歳）。全例矯正視力は1.0以上であった。以下に除外基準を述べる：-5D以上の近視、Emery-Little分類[10]3度以上の白内障、網膜疾患や黄斑疾患、網脈絡膜萎縮、ぶどう膜炎、緑内障、白内障手術を除く内眼手術の既往歴、5mm以下の散瞳不良例。77眼中に眼内レンズ挿入眼は21眼あった。

本研究はヘルシンキ宣言に則って行われ、藤田医科大学の倫理委員会の承認のもとで行われた。全ての被験者に本研究の目的、方法、研究に参加しない場合でも不利益のないことなどを説明した上で、被験者として研究に参加してもらう同意を得た。2015年のISCEVのガイドラインでは6種類のERGプロトコールが述べられている[1]。それらのERGの名称は暗順応か明順応か明記した後ろに刺激強度(単位：cd/$m^2$)で表記することになっている。本研究では6種類のうち4種類のERGプロトコール（暗順応0.01、暗順応3、明順応3、明順応フリッカERG）について検討した。0.5%フェニレフリンと0.5%トロピカミド点眼薬による散瞳をしてから20分間の暗順応の後、暗順応下のERGを記録した。その後10分間の明順応後に明順応下のERGを記録した。全ての反応は全視野刺激でERG記録装置(PuREC メイヨー、稲沢、日本)を使って記録した。刺激光と背景光の光源には白色LEDを用いた。

皮膚電極は銀皿電極を用い、関電極を下眼瞼、不関電極を外眼角、接

地電極を耳朶に置いた。バンドパスフィルターのローパスフィルターは500Hz、ハイパスフィルターは0.3Hzに設定し、10000倍に増幅した。反応の加算は暗順応下のERGでは10回、明順応下のERGでは32回から64回行った。PuRECに搭載されたPURE法を用いてノイズを除去した。

得られた波形からISCEVの標準化されたERGに示される各波形成分の振幅、潜時を計測した。a波の振幅は基線から窪みまで、b波の振幅はa波の窪みからb波の頂点までを計測した。暗順応0.01の振幅は基線から頂点まで、フリッカERGの振幅は窪みから頂点までを測定した。ISCEV extended protocol for the photopic negative response (PHNR)ではPhNRの記録には青色背景光下の赤色刺激が適しているとしている[11]が、明順応3ERGの反応からPhNRを測定している報告もある[12-18]。Ortizら[18]は明順応3ERGの波形にみられる2つのPhNRについてi波の前の窪みをPhNR1、i波の後ろの窪みをPhNR2として検討した。したがって本研究では明順応3ERGでのPhNR1をPhNRとしてその振幅を基線から窪みまでとした。各成分の潜時は刺激から波形の頂点までを計測した（図1左)。ISCEVの推奨[1]に従って振幅、潜時それぞれの中央値と$5^{th}$, $95^{th}$パーセンタイルを求めた上で、年齢との相関を線形単回帰分析（$p < 0.05$の場合を有意として標準化偏回帰係数を表記）を用いて検討した。

## 結果

皮膚電極から記録したISCEVの標準化されたERGの代表波形をPURE法の有無で比較して図1に示す。これは被験者の一人である22歳の女

性の右眼の反応である。最高矯正視力は右 1.0 x S-3.5D、左 1.0 x S-3.25D で、眼圧は両 12mmHg であった。前眼部、中間透光体、眼底に特に異常を認めなかった。暗順応下の ERG は明順応を避けるため、加算回数が少ない。PURE 法無しの場合、暗順応下の ERG ではノイズの混入が大きいため、振幅や潜時を測定することができなかったが、PURE 法を用いることでノイズの混入が小さくなったことが示されている。

表 1 に振幅の中央値と 5th, 95th パーセンタイル、年齢との線形単回帰分析の結果を示す。すべての成分で年齢との有意な負の相関を認めた。年齢と各成分の振幅の散布図と回帰直線を図 2 に示す。PhNR 以外の成分では弱い相関を示し、PhNR では中程度の相関を示した。(表 1)

表 2 に潜時の中央値と 5th, 95th パーセンタイル、年齢との線形単回帰分析の結果を示す。すべての成分で年齢との有意な正の相関を認めた。年齢と各成分の潜時の散布図と回帰直線を図 3 に示す。暗順応 0.01、暗順応 3 b 波、明順応 3 a 波、PhNR、明順応フリッカ ERG では弱い相関を示し、暗順応 3 a 波、明順応 3 b 波では中程度の相関を示した。(表 2)

考按

今回我々は PURE 法によるノイズ除去を用いることで皮膚電極からノイズの少ない良好な反応を記録することができた。これにより各成分の振幅と潜時の正常範囲を得ることができた。PURE 法は交流電源を参照しながら、フーリエ変換を用いてハムノイズを合成し、元波形から除去する。しかし、この方法でも眼瞼の筋電図の混入は除去できないため、平均加算回数を角膜電極で記

録する時の倍程度に増加させた。

年齢との相関については、全ての ERG 成分に対して振幅は有意な負の相関を示し、潜時は有意な正の相関を示した。この結果はこれまでの報告とほぼ一致していた。[2-7] PURE 法を用いることで皮膚電極でも、他の電極と同様に疾患の ERG を評価できる可能性がある。またその場合には年齢による振幅や潜時の変化も考慮すべきである。ERG で計測した振幅は網膜全体にわたる杆体細胞、錐体細胞、双極細胞、神経節細胞、アマクリン細胞、水平細胞、ミュラー細胞の細胞外電位変化の差し引きの結果である。[19, 20]加齢に伴う杆体細胞や双極細胞、神経節細胞、ミュラー細胞の減少[21-23]や、それに伴う signal transduction の変化が振幅の低下や潜時の延長の一因と考えられる。しかしそれぞれの細胞の変化を個別に捉えることは難しい。これには、より詳細な研究が必要である。

PhNR は網膜神経節細胞とその軸索の活動性を反映してしている。[24] ISCEV extended protocol for the PhNR では PhNR の記録にはより大きな振幅を得ることができるため、青色背景光下の赤色刺激が適しているとしている[11]が、今回我々は明順応 3 ERG の刺激条件である白色背景光下の白色刺激を用いた。で得られた波形の b 波に続く下向きの陰性波を PhNR として測定した。それでも青色背景光下の赤色刺激で測定した過去の報告[25]と同様に年齢と振幅は有意に負の相関を示し、潜時とは有意に正の相関を示した。

Miura らは grade2 や 3 の白内障患者から RETeval を用いて無散瞳で皮膚電極からフリッカ ERG を記録し、IOL 眼に比較して有意に振幅が低下、潜時が延長していることを報告した。[26] 今回の研究の対象者には Emery-Little

分類２度までの軽度の白内障は含まれているため、結果にも影響している可能性は否定できない。[27] しかしRETevalのフリッカERGの刺激に比べて、ISCEVの標準化されたERGではずっと強い刺激光を用いており、より強い刺激光では潜時は白内障の影響を受けにくい[28]ことから、今回の我々の結果へ軽度白内障の影響はより少ないと考える。またSuzukiらは25から91歳の網膜疾患のない31例の眼内レンズ挿入眼のS-cone ERGとLM-cone ERGを記録して、年齢による振幅の有意な減少と潜時の有意な延長を報告した[5]ことから、白内障の影響を除いた状態でも、加齢とERGの振幅や潜時は、今回と同様の相関があることがわかる。

今回の研究には以下の制限がある。（１）正常値を計測するにしては比較的症例数が少なかったため、より多数例において検討した場合には各ERG成分の振幅と潜時の中央値や5$^{th}$, 95$^{th}$パーセンタイルは変化しうる。（２）今回の研究の対象者には軽度の白内障は含まれているため白内障の影響を完全には排除できていない。理想的にはSuzukiら[5]のように対象を眼内レンズ挿入眼のみにすることであるが、若年者の眼内レンズ眼の症例数はさらに少ないため、データの取得が困難であることが予想される。

今回我々はPURE法によるノイズ除去を用いることで皮膚電極からノイズの少ない良好な波形を持つ正常者のISCEVの標準化されたERGを記録することができた。各ERG成分の年齢との相関は、振幅とは負の相関を、潜時とは正の相関を示した。今後は白内障の影響をさらに排除した多数例での検討が必要である。

謝辞

ERG 記録に際し技術的なサポートをしていただいた有限会社メイヨーの長坂英一郎氏、吉川眞男氏、工藤英貴氏に深謝します。

文献


1. McCulloch DL, Marmor MF, Brigell MG, Hamilton R, Holder GE, Tzekov R, Bach M. ISCEV Standard for full-field clinical electroretinography (2015 update). Doc Ophthalmol 2015; 130: 1-12.
2. Weleber RG. The effect of age on human cone and rod ganzfeld electroretinograms. Invest Ophthalmol Vis Sci 1981; 20: 392-9.
3. Wright CE, Williams DE, Drasdo N, Harding GF. The influence of age on the electroretinogram and visual evoked potential. Doc Ophthalmol 1985; 59: 365-84.
4. Birch DG, Anderson JL. Standardized full-field electroretinography. Normal values and their variation with age. Arch Ophthalmol 1992; 110: 1571-6.
5. Suzuki S, Horiguchi M, Tanikawa A, Miyake Y, Kondo M. Effect of age on short-wavelength sensitive cone electroretinogram and long- and middle-wavelength sensitive cone electroretinogram. Jpn J Ophthalmol 1998; 42: 424-30.
6. Kergoat H, Kergoat MJ, Justino L. Age-related changes in the flash electroretinogram and oscillatory potentials in individuals age 75 and older. J Am Geriatr Soc 2001; 49: 1212-7.
7. Parvaresh MM, Ghiasian L, Ghasemi Falavarjani K, Soltan Sanjari

M, Sadighi N. Normal values of standard full field electroretinography in an Iranian population. J Ophthalmic Vis Res 2009; 4: 97-101.

8. Dawson WW, Trick GL, Litzkow CA. Improved electrode for electroretinography. Invest Ophthalmol Vis Sci 1979; 18: 988-91.
9. Toda N. A cancellation method of periodic interference in pulse-like signals using adaptive filter and its apprication to flash ERGs. The IEICE Transactions on Information and Systems 2011; 94 1685-95(in Japanese).
10. Emery JM, Little JH. Phacoemulsification and aspiration of cataracts : surgical techniques, complications, and results. St. Louis: Mosby; 1979: 45-8.
11. Frishman L, Sustar M, Kremers J, McAnany JJ, Sarossy M, Tzekov R, Viswanathan S. ISCEV extended protocol for the photopic negative response (PhNR) of the full-field electroretinogram. Doc Ophthalmol 2018; 136: 207-11.
12. Machida S, Gotoh Y, Tanaka M, Tazawa Y. Predominant loss of the photopic negative response in central retinal artery occlusion. Am J Ophthalmol 2004; 137: 938-40.
13. Gotoh Y, Machida S, Tazawa Y. Selective loss of the photopic negative response in patients with optic nerve atrophy. Arch Ophthalmol 2004; 122: 341-6.

14. Kizawa J, Machida S, Kobayashi T, Gotoh Y, Kurosaka D. Changes of oscillatory potentials and photopic negative response in patients with early diabetic retinopathy. Jpn J Ophthalmol 2006; 50: 367-73.
15. Ueno S, Kondo M, Piao CH, Ikenoya K, Miyake Y, Terasaki H. Selective amplitude reduction of the PhNR after macular hole surgery: ganglion cell damage related to ICG-assisted ILM peeling and gas tamponade. Invest Ophthalmol Vis Sci 2006; 47: 3545-9.
16. Miyata K, Nakamura M, Kondo M, Lin J, Ueno S, Miyake Y, Terasaki H. Reduction of oscillatory potentials and photopic negative response in patients with autosomal dominant optic atrophy with OPA1 mutations. Invest Ophthalmol Vis Sci 2007; 48: 820-4.
17. Thompson DA, Feather S, Stanescu HC, Freudenthal B, Zdebik AA, Warth R, Ognjanovic M, Hulton SA, Wassmer E, Van'T Hoff W, Russell-Eggitt I, Dobbie A, Sheridan E, Kleta R, Bockenhauer D. Altered electroretinograms in patients with KCNJ10 mutations and EAST syndrome. JPhysiol 2011; 589: 1681-9.
18. Ortiz G, Drucker D, Hyde C, Staffetti J, Kremers J, Tzekov R. The photopic negative response of the Light-adapted 3.0 ERG in clinical settings. Doc Ophthalmol 2020; 140: 115-28.

19. Bush RA, Sieving PA. A proximal retinal component in the primate photopic ERG a-wave. Invest Ophthalmol Vis Sci 1994; 35: 635-45.
20. Sieving PA, Murayama K, Naarendorp F. Push-pull model of the primate photopic electroretinogram: a role for hyperpolarizing neurons in shaping the b-wave. Vis Neurosci 1994; 11: 519-32.
21. Dorey CK, Wu G, Ebenstein D, Garsd A, Weiter JJ. Cell loss in the aging retina. Relationship to lipofuscin accumulation and macular degeneration. Invest Ophthalmol Vis Sci 1989; 30: 1691-9.
22. Gao H, Hollyfield JG. Aging of the human retina. Differential loss of neurons and retinal pigment epithelial cells. Invest Ophthalmol Vis Sci 1992; 33: 1-17.
23. Curcio CA, Millican CL, Allen KA, Kalina RE. Aging of the human photoreceptor mosaic: evidence for selective vulnerability of rods in central retina. Invest Ophthalmol Vis Sci 1993; 34: 3278-96.
24. Viswanathan S, Frishman LJ, Robson JG, Harwerth RS, Smith EL, 3rd. The photopic negative response of the macaque electroretinogram: reduction by experimental glaucoma. Invest Ophthalmol Vis Sci 1999; 40: 1124-36.
25. Joshi NR, Ly E, Viswanathan S. Intensity response function of the photopic negative response (PhNR): effect of age and test-retest

reliability. Doc Ophthalmol 2017; 135: 1-16.

26. Miura G, Nakamura Y, Sato E, Yamamoto S. Effects of cataracts on flicker electroretinograms recorded with RETeval system: new mydriasis-free ERG device. BMC Ophthalmol 2016; 16: 22.

27. Tanikawa A, Suzuki K, Nomura R, Tanaka H, Mizuguchi T, Shimada Y, Horiguchi M. The influence of mild cataract on ISCEV standard electroretinogram recorded from mydriatic eyes. Doc Ophthalmol 2020 (in press).

28. Miura G, Sato E, Yamamoto S. Flicker electroretinograms recorded with mydriasis-free RETeval system before and after cataract surgery. Eye (Lond) 2017; 31: 1589-93.

## 図の説明

**図 1. ERG の代表波形と pulse reference power line noise reduction（PURE）の有無による比較**

眼科疾患のない22歳女性の右眼の反応である。PURE法なしの暗順応下のERGではノイズが大きいため振幅や潜時が計測できなかったが、PURE 法を用いるとノイズは比較的小さかった。DA: Dark adapted, LA: Light adapted

矢頭：刺激、★：a 波、☆：b 波、◆：photopic negative response

**図 2. ISCEV の標準化された ERG の各成分の振幅と年齢の散布図**

DA: Dark adapted, LA: Light adapted, PhNR: photopic negative response

すべての成分で線形単回帰分析で有意な負の相関がみられ、その回帰直線をグラフ内に示した。相関係数や p 値は表 1 に示した。

**図 3. ISCEV の標準化された ERG の各成分の潜時と年齢の散布図**

DA: Dark adapted, LA: Light adapted, PhNR: photopic negative response

すべての成分で線形単回帰分析で有意な正の相関がみられ、その回帰直線をグラフ内に示した。相関係数や p 値は表 2 に示した。

**表 1. 振幅の中央値と $5^{th}$, $95^{th}$ パーセンタイル、年齢との相関**

DA: Dark adapted, LA: Light adapted, PhNR: photopic negative response

**表 2. 潜時の中央値と 5th, 95th パーセンタイル、年齢との相関**

DA: Dark adapted, LA: Light adapted, PhNR: photopic negative response
